# Alkaline, Neutral and Acidic Extracts from Rubber Tree (*Hevea brasiliensis*) Seed Shells and Their Application as Biodiesel Antioxidants

**DOI:** 10.1021/acsomega.6c01549

**Published:** 2026-05-11

**Authors:** Giovanna Oleinik, Giovano Tochetto, Letiére C. Soares, Fernanda O. Lima, Dalila M. Benvegnú, Gabrielle C. Peiter, André L. Gallina

**Affiliations:** 1 Department of Chemistry, Midwestern Paraná State University (UNICENTRO), Alameda Élio Antonio Dalla Vecchia, 838, Center for Technological Development of Guarapuava, Paraná (PR) 85040-167, Brazil; 2 232192 Federal University of Fronteira Sul (UFFS), Campus Realeza, Postal Office Box 253, Avenida Edmundo Gaievski, 1000, Rodovia BR 182 − Km 466, Realeza, Paraná (PR) 85770-000, Brazil; 3 Graduate Program in Chemical and Biotechnological Processes, Federal University of Technology - Paraná (UTFPR), Rua Cristo Rei, 19, Toledo, Paraná (PR) 85902-490, Brazil

## Abstract

Biodiesel is a viable alternative to petroleum diesel, with several advantages, including lower pollutant emissions. However, biodiesel possesses a low oxidation stability due to its composition, which complicates its transport, storage, and consumption. Thus, the addition of antioxidants is mandatory, and most of the antioxidants used are synthetic, creating a need for the development of new natural antioxidant feedstocks. In this context, the rubber tree (*Hevea brasiliensis*) seed is a coproduct of the rubber tree cultivation, which is widely used for the extraction of latex. These seeds do not currently possess large-scale applications. Therefore, the objective of this study was to produce acidic, alkaline, and neutral medium extracts from rubber tree seed shells to be used as antioxidant additives to soybean oil biodiesel. The extraction processes were optimized via mathematical modeling, the antioxidant activity of the extracts was quantified with the DPPH (2,2-diphenyl-1-picrylhydrazyl) capture method, and total phenolic compounds were quantified via the Folin–Ciocalteau method. Furthermore, the extracts were added to biodiesel, and its oxidation stability was quantified using a Rancimat 873 device. In accordance, the acid medium extract reached 79.664% antioxidant activity and increased the induction time to 16.29 ± 0.14 h when added in combination with 100 ppm of tertiary butylhydroquinone (TBHQ), indicating synergistic effects. Conversely, in conjunction with 100 ppm of TBHQ, the alkaline and neutral extracts reduced the induction time from 5.00 ± 0.10 h (control) to 3.11 ± 0.02 h and 2.31 ± 0.06 h, respectively. Moreover, the biodiesel with acidic extract possessed characteristics in accordance with the Brazilian National Petroleum, Natural Gas, and Biofuels Agency reference values. Accordingly, it was concluded that the rubber tree seed shell acidic medium extract possesses strong synergy with TBHQ, and mixes of the two are viable as antioxidants for soybean-based biodiesel.

## Graphical abstract

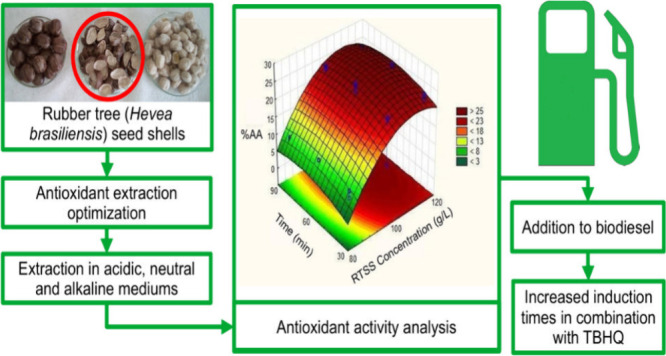

## 1 Introduction

Currently, fossil fuels supply most of the world’s growing energy demand.^1^ However, these nonrenewable sources cause severe environmental damage, including the release of greenhouse gases linked to climate change and adverse health effects.^2–4^ Consequently, research into the renewable biofuels, such as biodiesel and ethanol, has intensified. These alternatives offer a promising solution to fuel depletion and pollution while promoting global sustainable development.^5,6^

In this context, biodiesel is a promising biofuel that is especially useful as a substitute for diesel. Chemically, biodiesel is a mixture of long-chain fatty esters, typically produced through the transesterification of various lipid sources, such as vegetable oils or animal fats.^7,8^ Furthermore, this biofuel possesses a largely closed carbon cycle. The plants used in its production absorb carbon dioxide (CO_2_) from the atmosphere during photosynthesis, resulting in lower net CO_2_ emissions than conventional diesel.^2,9^

However, biodiesel is prone to oxidative degradation due to the presence of unsaturated fatty acids, which are highly susceptible to oxidation.^10^ This process can be accelerated by factors such as high temperatures and exposure to oxygen, light, water, or metals.^2,7^ Consequently, oxidation products are formed, including hydroperoxides, acids, aldehydes, and ketones. These products degrade biodiesel quality and increase the risk of engine corrosion, injector nozzle fouling, filter clogging, and carbon deposition, while also complicating long-term storage.^5,11^ To address this, a common method to increase the oxidative stability of biodiesel is the addition of antioxidant compounds that inhibit these processes.^5^

Antioxidants are effective even at very low concentrations. They function by donating hydrogen atoms to free radicals or reactive oxygen species, thereby inhibiting the oxidation pathway.^5,12^ Currently, both synthetic and natural antioxidants are added to biodiesel, with synthetic variants, butylated hydroxytoluene (BHT), *tert*-butylhydroquinone (TBHQ), butylated hydroxyanisole (BHA), pyrogallol (PY), and propyl gallate (PG), being the most prevalent in the industry. However, these synthetic additives increase production costs and are inherently unsustainable, as they are primarily petroleum-derived. Furthermore, their potential toxicity and carcinogenicity pose significant health risks.^2,5,7^

Therefore, to reduce costs and negative environmental impacts, research into natural and renewable antioxidants as substitutes for synthetic ones in biodiesel has increased considerably in recent years.^2,5,7^ Furthermore, plants contain phenolic compounds (secondary metabolites with easily extractable hydrogen atoms) that can stabilize free radicals during oxidation by hydrogen donation, thus offering a promising alternative to petroleum-derived antioxidants.^10,11^

In this context, the rubber tree (*Hevea brasiliensis*) seed represents a viable natural feedstock for producing antioxidant additives for biodiesel. A member of the *Euphorbiaceae* family native to the Amazon region, the rubber tree is primarily cultivated for latex extraction. Consequently, its seeds have few industrial applications, and are often discarded despite being produced at rates of 70 to 500 kg per hectare annually.^13–15^ Developing profitable uses for this coproduct is therefore highly justified. For instance, Lüneburger et al.^16^ treated rubber tree seed oil to reduce acidity for biodiesel production, while Tochetto et al.^17^ reported on the acid hydrolysis and ethanol production from rubber tree seed kernels. Moreover, Baidoo et al.^15^ used *Hevea brasiliensis* seed oil for the production of sustainable aviation biofuel. Despite these applications, research into the antioxidant properties of the seeds remains limited, with only a few studies, such as that by Oleinik et al.,^18^ who evaluated the antioxidant activity of extracts from defatted rubber tree seed kernels.

Thus, the antioxidant capacity of rubber tree seed shells (RTSS) warrants further study, given the current scarcity of research in this area. Considering the demand for sustainable, natural antioxidants that minimize environmental and health impacts, the objective of this study was to evaluate the efficacy of acidic, alkaline, and neutral extracts from *Hevea brasiliensis* seed shells in inhibiting the oxidation of soybean-based biodiesel.

## 2 Results and Discussion

### 2.1 Determination and Optimization of the Extracts’ Antioxidant Activity

Table 1 presents the results for the antioxidant extraction assays. All experimental runs were performed in triplicate to allow for the estimation of pure error and to minimize the lack-of-fit in the regression models. Note that for the neutral extract optimization, triplicates were conducted exclusively at the center points of the design space to establish baseline reproducibility.

**Table 1:** Experimental Design and Observed Responses for the Optimization of Antioxidant Extraction in Different Media

independent variables		obtained antioxidant activity (%)		
time	concentration	alkaline medium	neutral medium	acidic medium
–1	–1	8.64 ± 1.17	48.99	66.76 ± 0.63
–1	0	9.90 ± 0.92	47.97	79.02 ± 0.47
–1	1	9.33 ± 0.46	54.38	73.44 ± 2.26
0	–1	18.84 ± 7.51	51.49	42.99 ± 0.15
0	0	25.00 ± 1.28	59.80 ± 0.83	59.49 ± 0.36
0	1	23.83 ± 0.47	58.36	62.52 ± 0.83
1	–1	23.61 ± 0.94	42.91	13.44 ± 2.31
1	0	24.57 ± 1.66	44.08	39.18 ± 1.38
1	1	24.18 ± 0.08	48.51	25.95 ± 0.35

#### 2.1.1 Alkaline Medium Extracts

As described in Section 4.2, antioxidant extraction optimization in an alkaline medium were carried out according to the 3^2^ full factorial experimental design. From these results, a quadratic mathematical model was developed, as shown in eq 1.

$$\%\mathrm{AA}=23.72+7.42c-5.85c^{2}+1.04t-1.75^{2}-0.03\mathrm{c t}1$$ 1

In which %AA represents the percentage of antioxidant activity, c represents the RTSS concentration and t represents the extraction time.

Furthermore, the model’s ANOVA is presented in Table 2. The time variable was statistically significant (*p* < 0.05) in both its linear and quadratic terms, whereas the concentration variable and the interaction between variables were not (*p* > 0.05), as also depicted in the Pareto graph of Figure S1. The model yielded a coefficient of determination (R^2^) of 0.8858, accounting for 88.58% of the explainable variance. This is a high degree of fit, nearly reaching the 90.74% maximum explainable limit dictated by experimental error. Furthermore, the F-ratio value (32.58) significantly exceeded the tabulated F value (Ftab = 2.68 at 5 and 21 degrees of freedom), confirming the model’s adequacy. The lack-of-fit test was nonsignificant (FLoF = 1.40 < Ftab = 3.16 at 3 and 18 degrees of freedom), and the randomly dispersed residuals shown in Figure S2 confirm that the quadratic model is statistically sound.^19,20^

**Table 2:** ANOVA of the Mathematical Model for the Alkaline Medium Antioxidant Extraction Optimization

**factor** ^a^	**SS** ^b^	**df** ^c^	**MS^d^**	*F*	*p*
regression (R)	1233.12	5	246.62	32.58	<0.05
time (L)	989.68	1	989.68	138.24	0.000000
time (Q)	205.53	1	205.53	28.71	0.000043
concentration (L)	19.53	1	19.53	2.73	0.115932
concentration (Q)	18.36	1	18.36	2.57	0.126657
time (L) × concentration (L)	0.013	1	0.013	0.001	0.966053
residuals (r)	158.94	21	7.57		
lack of Fit (LoF)	30.08	3	10.03	1.40	0.275124
pure error (PE)	128.87	18	7.16		
total SS	1392.06	26			

a L: linear; Q: quadratic.

b SS: square sum.

c df: degrees of freedom.

d MS: mean of squares.

Figure 1 presents the response surface plot for the alkaline medium extraction optimization. As shown, the optimum region was identified between 60 and 90 min and between 100 and 120 g·L^–1^. Furthermore, the specific optimum point was determined using the desirability function, as shown in Figure 2. Based on this optimization, the highest antioxidant activity, predicted to be 26.048%, would be obtained at a concentration of 112 g·L^–1^ and an extraction time of 78 min.

**Figure 1:**
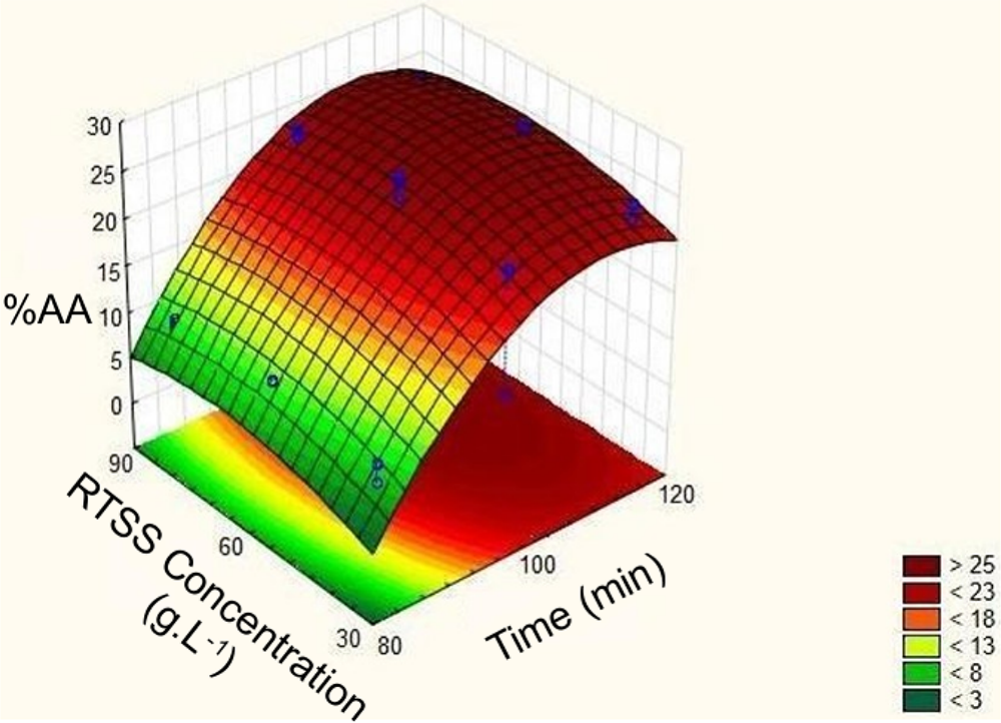
Response surface of the alkaline medium extraction optimization.

**Figure 2:**
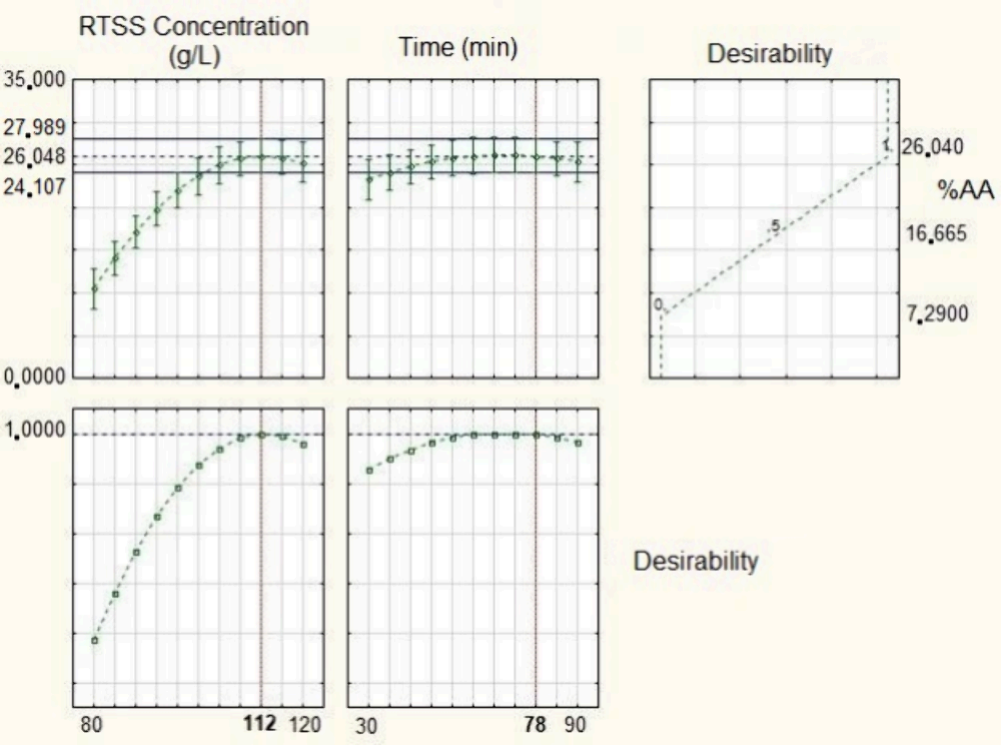
Alkaline medium extraction desirability graphs.

Finally, the validation trial for the optimized point of the selected quadratic model yielded an experimental result of 27.453 ± 0.313%, compared to a predicted value of 26.048%. While a one-sample *t* test indicated a statistical difference (*p* < 0.05), as detailed in Table S1, the Relative Error (RE) was only 5.38%, and the experimental result remained within the 95% confidence interval (24.107–27.989%) shown in Figure 2. This statistical significance is likely a result of the high precision (low standard deviation) of the experimental replicates, which inherently increases the sensitivity of the *t* test. Given the low RE and the alignment with the confidence interval, the model is considered a functionally accurate tool for predicting antioxidant activity in this medium.

#### 2.1.2 Neutral Medium Extracts

As described in Section 4.2, antioxidant extraction optimization in a neutral medium was carried out according to the 3^2^ full factorial experimental design. From these results, a quadratic mathematical model was developed, as shown in eq 2.

$$\%\mathrm{AA}=58.21-2.64t-9.80t^{2}+2.97c-0.90^{2}+0.05\mathrm{t c}2$$ 2

The model’s ANOVA is presented in Table 3. Both the linear and quadratic terms for the time variable were statistically significant (*p* < 0.05), while the concentration variable was significant only in its linear term (*p* < 0.05). The interaction between time and concentration, however, was not statistically significant (*p* > 0.05), as also depicted in the Pareto graph of Figure S3. The model yielded a R^2^ of 0.9042, accounting for 90.42% of the explainable variance. This is a high degree of fit, nearly reaching the 99.67% maximum explainable limit dictated by experimental error. Furthermore, the F-ratio value (9.43) exceeded the tabulated F value (Ftab = 5.05 at 5 and 5 degrees of freedom), confirming the model’s adequacy. The lack-of-fit test was nonsignificant (FLoF = 18.39 < Ftab = 19.16 at 3 and 2 degrees of freedom), and the randomly dispersed residuals shown in Figure S4 further validates the quadratic model.^19,20^

**Table 3:** ANOVA of the Mathematical Model for the Neutral Medium Antioxidant Extraction Optimization

**factor** ^a^	**SS** ^b^	**df** ^c^	MS^d^	*F*	*p*
regression (R)	372.15	5	74.43	9.43	<0.05
time (L)	41.79	1	41.79	60.97	0.016008
time (Q)	243.42	1	243.42	355.12	0.002804
concentration (L)	53.16	1	53.16	77.55	0.012651
concentration (Q)	2.07	1	2.07	3.02	0.224180
time (L) × concentration (L)	0.0108	1	0.0108	0.02	0.911623
residuals (r)	39.45	5	7.89		
lack of fit (LoF)	38.08	3	12.69	18.52	0.051669
pure error (PE)	1.37	2	0.69		
total SS	411.60	10			

a L: linear; Q: quadratic.

b SS: square sum.

c df: degrees of freedom.

d MS: mean of squares.

Figure 3 displays the response surface for the neutral medium extraction optimization. As shown, an optimum region was reached at an extraction time of approximately 150 min and a RTSS concentration of nearly 180 g·L^–1^. Furthermore, the specific optimum point was determined using the desirability function, as shown in Figure 4. Based on this analysis, the highest predicted antioxidant activity (60.41%) would be obtained at a concentration of 180 g·L^–1^ and an extraction time of 144 min.

**Figure 3:**
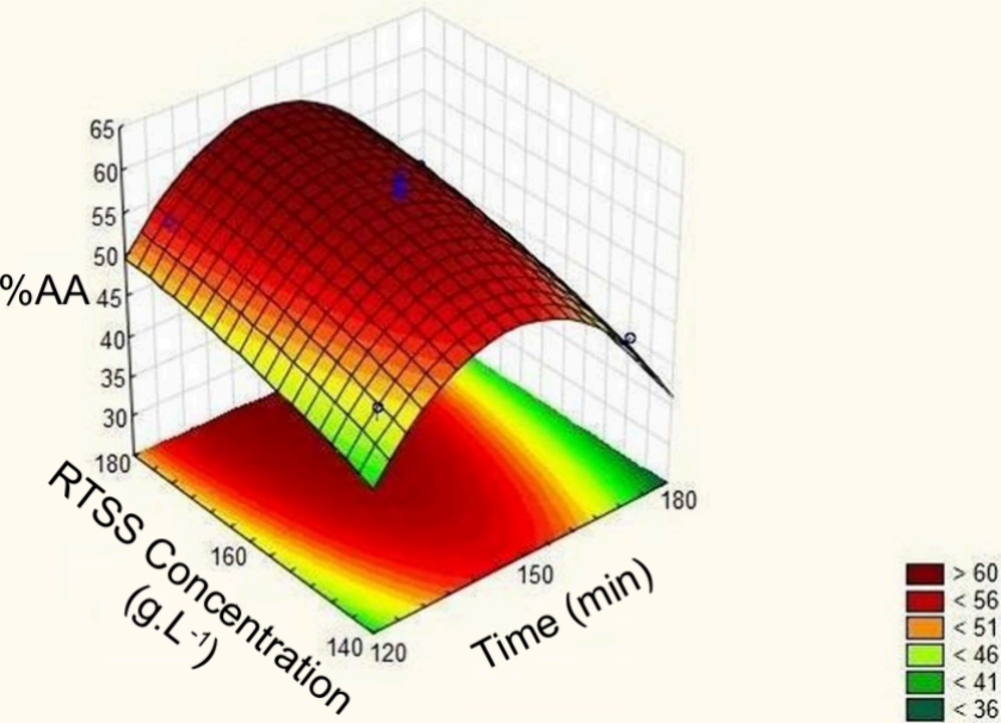
Response surface of the neutral medium extraction optimization.

**Figure 4:**
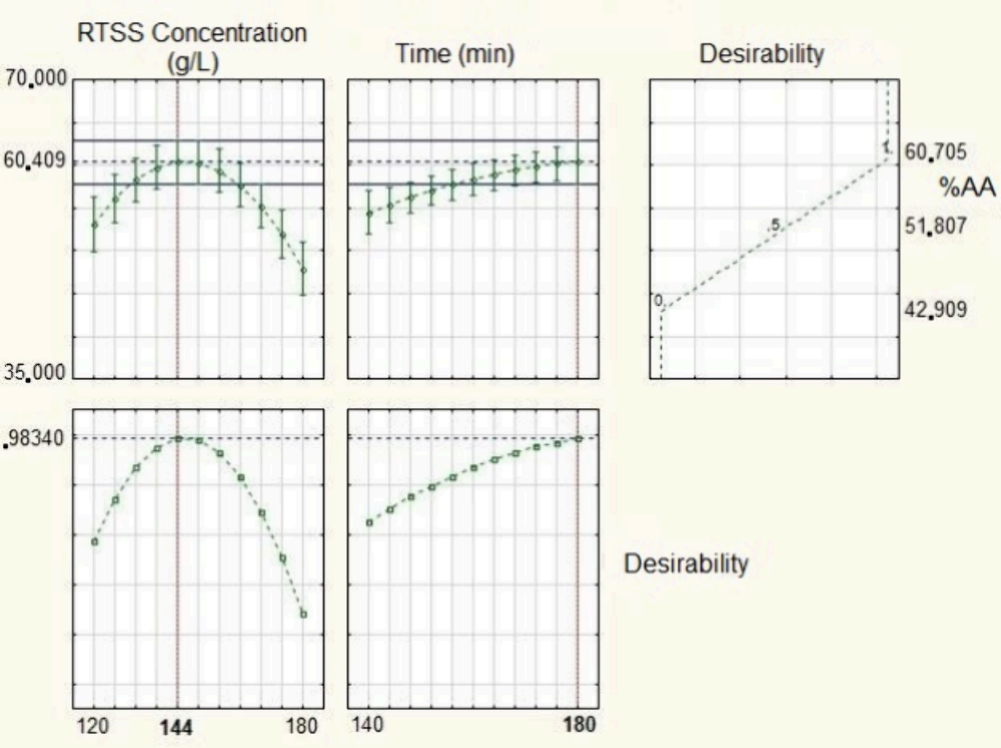
Neutral medium extraction desirability graphs.

To validate the model’s predictive capability across the entire experimental design space, validation tests were performed at the boundaries of the domain. At the codified coordinates of (−1; 1), representing an edge point for time and concentration respectively, an experimental result of 54.38 ± 0.56% was observed, compared to a predicted value of 53.07% (calculated with eq 2). As detailed in Supplementary Table S2, a one-sample *t* test confirmed no statistical difference between these values (*p* > 0.05), indicating that the model’s predictive power is not restricted to the center of the domain, but is valid throughout the studied range.

#### 2.1.3 Acidic Medium Extracts

As described in Section 4.2, antioxidant extraction optimization in an acidic medium was carried out according to the 3^2^ full factorial experimental design. From these results, a quadratic mathematical model was developed, as shown in eq 3.

$$\%\mathrm{AA}=62.81-23.44t-5.37t^{2}+6.45c-11.71c^{2}+1.46\mathrm{t c}$$ 3

In which c represents the RTSS concentration and t represents the extraction time.

The model’s ANOVA is presented in Table 4. Both independent variables and their interaction were statistically significant (*p* < 0.05) in their linear and quadratic terms, as also depicted in the Pareto graph of Figure S5. The model yielded a R^2^ of 0.9739, accounting for 97.39% of the explainable variance. Furthermore, the F-ratio value (156.65) significantly exceeded the tabulated F value (Ftab = 2.68 at 5 and 21 degrees of freedom), confirming the model’s adequacy. Although the lack-of-fit test was significant (FLoF = 61.24 > Ftab = 3.16), this result may be attributed to the exceptionally low experimental ‘pure error.’ The Pure Error Sum of Squares (27.87) represents only 0.23% of the Total Sum of Squares (Total SS = 11975.26), a level of precision that inherently renders the F-test highly sensitive to even minor model deviations. However, the model’s overall significance is confirmed by a high F-ratio of 156.65 (nearly 60 times the tabulated value) and a coefficient of determination (R^2^) of 0.9739. Combined with the randomly dispersed residuals shown in Figure S6, these parameters indicate that the quadratic model remains a statistically robust and reasonably accurate tool for identifying the optimized extraction conditions.^19,20^

**Table 4:** ANOVA of the Mathematical Model for the Acidic Medium Antioxidant Extraction Optimization

**factor** ^a^	**SS** ^b^	**df** ^c^	**MS** ^d^	*F*	*p*
regression (R)	11662.63	5	2332.53	156.65	<0.05
time (L)	9891.60	1	9891.60	6389.06	0.000000
time (Q)	172.75	1	172.75	111.58	0.000000
concentration (L)	749.39	1	749.39	484.03	0.000000
concentration (Q)	823.43	1	823.43	531.86	0.000000
time (L) × concentration (L)	25.47	1	25.47	16.45	0.000741
residuals (r)	312.63	21	14.89		
lack of fit (LoF)	284.76	3	94.92	61.31	0.000000
pure error (PE)	27.87	18	1.55		
total SS	11975.26	26			

a L: linear; Q: quadratic.

b SS: square sum.

c df: degrees of freedom.

d MS: mean of squares.

Figure 5 presents the response surface for the acidic medium extraction optimization optimization. As shown, the optimum region was observed at an extraction time of approximately 60 min and a RTSS concentration between 100 and 110 g·L^–1^. Moreover, the specific optimum point was determined using the desirability function, as shown in Figure 6. Based on this analysis, the highest predicted antioxidant activity (79.66%) would be obtained at a concentration of 106 g·L^–1^ and an extraction time of 60 min.

**Figure 5:**
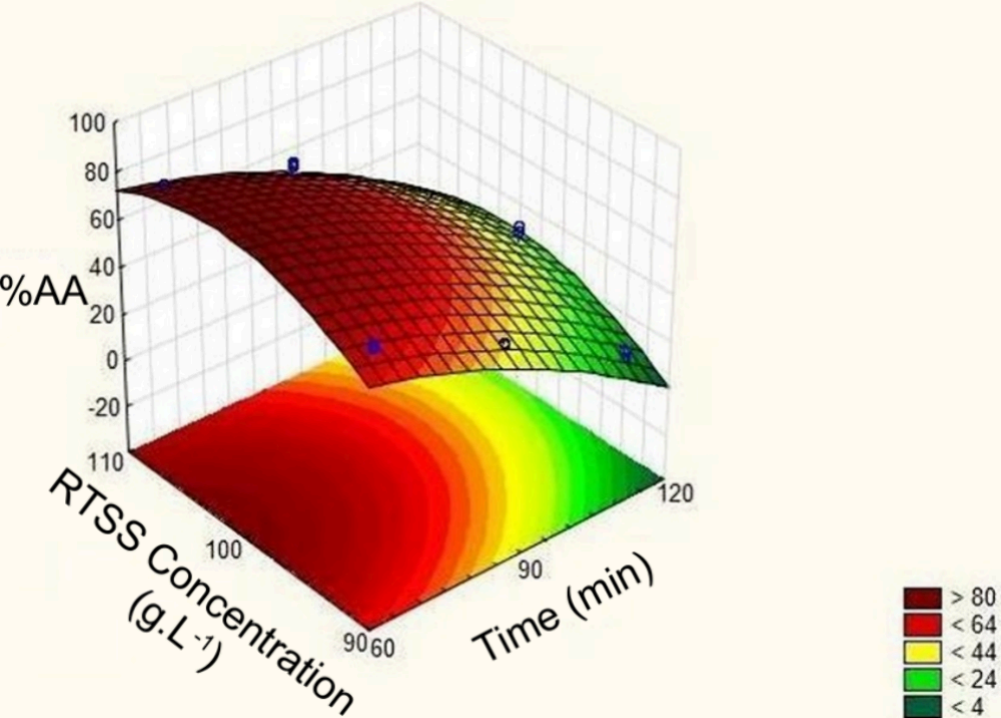
Response surface of the acidic medium extraction optimization.

**Figure 6:**
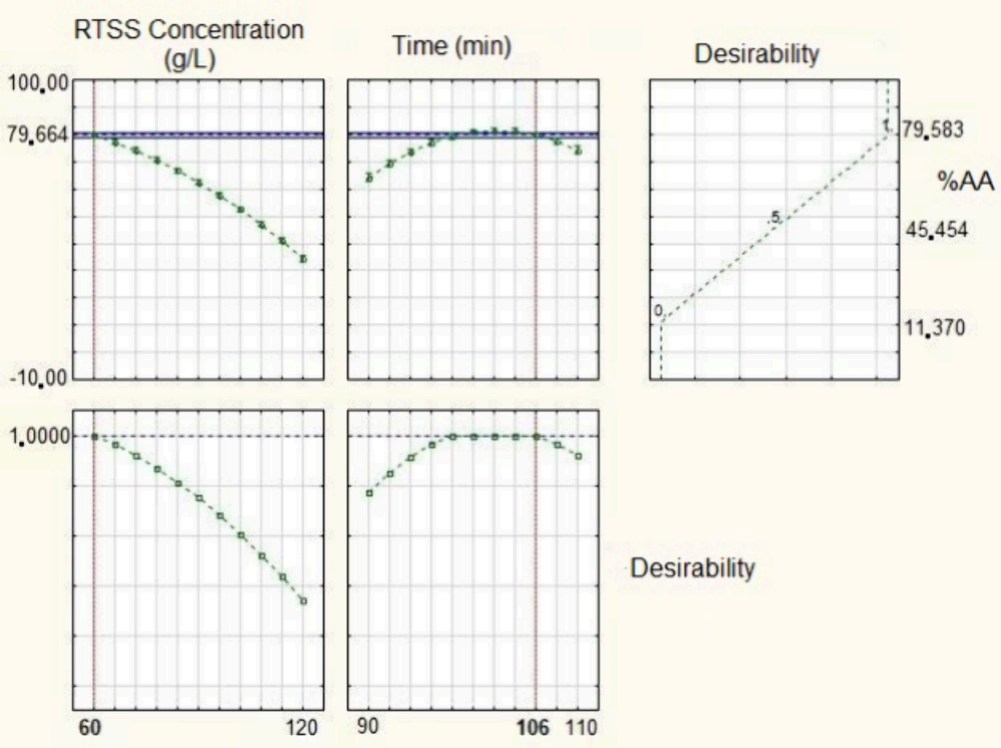
Acidic medium extraction desirability graphs.

To validate the model’s predictive capability across the entire experimental design space, validation tests were performed at the boundaries of the domain. At the codified coordinates of (−1; 1), representing an edge point for time and concentration respectively an experimental result of 73.44 ± 2.26% was observed, compared to a predicted value of 74.16% (calculated with eq 3). As detailed in Supplementary Table S3, a one-sample *t* test confirmed no statistical difference between these values (*p* > 0.05), indicating that the model’s predictive power is not restricted to the center of the domain, but is valid throughout the studied range.

#### 2.1.4 Comparison with Other Works

Following the classification established by Sousa et al.,^21^ a 2,2-diphenyl-1-picrylhydrazyl (DPPH) radical scavenging activity above 70% may be considered strong. Values between 50% and 70% are categorized as moderate, while those below 50% indicate weak antioxidant activity. Based on these criteria, the acidic extract (79.664%) exhibits strong activity, the neutral extract (60.409%) shows moderate activity, and the alkaline extract (26.048%) is classified as weak.

Currently, few studies evaluate the radical scavenging capacity of natural extracts produced with alkaline or acidic solvents. Consequently, the results for the neutral methanolic extract are more readily comparable to existing literature. The present study’s result (60.409%), aligns with the findings of Bera et al.,^22^ who reported a 64.21 ± 0.7% antioxidant activity for the hydromethanolic extract of *Swietenia maagoni* (L.) Jacq. Seeds at 100 μg.mL^–1^. Similarly, Iqbal et al.^23^ observed a 64.28 ± 4.49% activity for *Datura stramonium* L. seed methanolic extracts at 60 μg.mL^–1^. Furthermore, Noipha et al.^24^ reported a slightly higher activity of 71.2 ± 0.17% for *Hevea brasiliensis* leaf extracts, at 500 μg.mL^–1^, using 95% ethanol as the solvent.

Furthermore, the results of the present study surpass those reported by Yesiloglu et al.,^25^ who observed a 36.1% scavenging activity for the methanolic extract of *Zingiber officinale* L. seeds at 250 μg.mL^–1^. Similarly, the observed values are significantly higher than those of Uddin et al.^26^ whose analysis of *Baccaurea ramiflora* Lour. methanolic extracts yielded an antioxidant activity of 32.67 ± 0.99% at 200 μg.mL^–1^.

Furthermore, the superior antioxidant activity of the acidic extract observed in this study may be attributed to the fact that acidic solvents favor the extraction of basic compounds, as their protonation increases their solubility. Second, antioxidant compounds with a basic character tend to exhibit greater antioxidant activity in the DPPH test. Supporting this, Pękal and Pyrzynska^27^ studied the antioxidant activity of tea infusions extracted with water, and found that the greatest antioxidant activities were observed for infusions with higher pH, indicating that alkaline compounds were extracted.

Given that the reaction of an antioxidant compound with DPPH involves proton donation from the antioxidant to the free radical, reducing it to DPPH-H,^12^ it follows that extracting antioxidants in an alkaline medium results in lower antioxidant activities. In these conditions, the antioxidant compounds undergo deprotonation within the alkaline solvent, rendering them unavailable for the subsequent DPPH reaction. Conversely, when extracted with acidic solvents, these compounds retain their protons, which remain available for donation to free radicals.^28,29^

### 2.2 Total Phenolic Compounds Quantification and Ultrahigh-Performance Liquid Chromatography (UHPLC) Phenolic Compound Profiles

The total phenolic compounds of the optimized extracts was quantified using the Folin-Ciocalteau method. Analysis revealed that the acidic extract contained 761.15 ± 116.23 mg GAE·100 g^–1^, while the neutral and alkaline extracts possessed 1043.77 ± 35.71 and 412.61 ± 47.83 mg GAE·100 g^–1^, respectively. These results corroborate the optimization findings, as the higher phenolic compound values in the neutral and acidic extracts align with their superior antioxidant performance compared to the alkaline medium. This relationship may be attributed to the inherent scavenging properties of phenolic compounds.^30–32^ Supporting this, Oleinik et al.^18^ demonstrated that antioxidant activity is directly proportional to phenolic concentration in rubber tree seed bagasse extracts.

Due to its highest phenolic concentration, the optimized neutral extract was selected for comparison with existing literature. The observed phenolics values aligns with the results of Uddin et al.^26^ who reported 1296 ± 0.77 mg GAE·100 g^–1^ for *Baccaurea ramiflora* Lour. seed methanolic extracts (1,000 μg.mL^–1^). Similarly, Oleinik et al.^18^ prepared methanolic extracts from *Hevea brasiliensis* seed bagasse at a concentration of 40 g·L^–1^ and obtained 1539.7 mg GAE·100 g^–1^. Meanwhile, Tlili et al.^33^ found 814 mg GAE·100 g^–1^ in *Capparis spinosa* seed extracts. Furthermore, Weidner et al.^34^ prepared extracts from *in natura* seeds of three species, namely, *V. amurensis*, *V. california*, and *V. riparia*. They reported 920 ± 0.2 mg·100 g^–1^, 1750 ± 0.4 mg·100 g^–1^ and 1970 ± 0.7 mg·100 g^–1^ of total phenolics, respectively. Also, Weidner et al.^35^ prepared extracts from *Vitis coignetiae* and *Vitis vinifera* seeds and found total phenolic contents of 899 ± 0.06 mg·100 g^–1^ and 2121 ± 0.05 mg·100 g^–1^, respectively. These comparisons demonstrate that the phenolic content of the optimized extracts in this study is consistent with other high-potency natural feedstocks.

Figures S7–S9 present the chromatographic profiles of the alkaline, neutral and acidic extracts, respectively. Chromatographic analysis of the neutral extract (Figure S8) confirms the presence of caffeic acid, while the acidic extract (Figure S9) reveals both caffeic and ellagic acids. These findings align with existing literature. For instance, caffeic acid has been widely reported in various nut species,^36^ and its presence in methanolic extracts of *Coffea arabica* seeds has also been documented.^37^ Similarly, the identification of ellagic acid is consistent with findings in pecan nut extracts^38^ and *Cucumbis melo* L. seed extracts,^39^ both of which exhibit phenolic profiles comparable to rubber tree seeds. These results suggest that the extraction method significantly influences the specific phenolic compounds recovered, with acidic conditions favoring the coextraction of caffeic and ellagic acids.

Furthermore, as shown in Figure S7, no phenolic compounds were identified in the alkaline extract, a result consistent with the lower phenolic compound values and diminished antioxidant activity observed in this medium. Finally, multiple unidentified peaks were detected in both the neutral and acidic extract chromatograms. These peaks likely represent additional phenolic compounds; however, they remained unidentified due to the absence of corresponding analytical standards.

### 2.3 Evaluation of the Antioxidant Effect over Biodiesel

Table 5 presents the induction times for biodiesel samples treated with optimized antioxidant extracts, homogenized via mechanical or ultrasonic agitation, compared to a control sample without additives. Notably, none of the biodiesel samples reached the minimum 13-h induction time required by ANP N°920/2023.^40^ Furthermore, only the acidic extracts yielded an improved induction time compared to the control (3.65 ± 0.12 h). Specifically, the mechanically agitated acidic extract achieved 6.03 ± 0.04 h, and the ultrasound-homogenized acidic extract reached 5.09 ± 0.16 h.

**Table 5:** Induction Times of Biodiesel Samples, Both Pure (control) and Antioxidant Extract Added

sample	induction time (h)
biodiesel transesterified with mechanical agitation	3.65 ± 0.12
biodiesel transesterified with ultrasound agitation	2.67 ± 0.02
biodiesel + alkaline extract (added by mechanical agitation)	3.7 ± 0.00
biodiesel + alkaline extract (added by ultrasound agitation)	2.07 ± 0.00
biodiesel + neutral extract (added by mechanical agitation)	1.15 ± 0.00
biodiesel + neutral extract (added by ultrasound agitation)	2.07 ± 0.06
biodiesel + acidic extract (added by mechanical agitation)	6.03 ± 0.04
biodiesel + acidic extract (added by ultrasound agitation)	5.09 ± 0.16

Given the modest induction times of the individual antioxidant extracts, further tests were conducted by adding 100 ppm TBHQ to biodiesel and biodiesel with extract samples. As shown in Table 6, both the alkaline and neutral extract samples yielded induction times than the TBHQ control (5.00 ± 0.10 h). Conversely, the acidic extract samples, added via both mechanical and ultrasonic agitation, exhibited significantly higher induction times, indicating a potentiation synergistic effect between the extracts and the synthetic antioxidant. Notably, the biodiesel with acidic extract homogenized via mechanical agitation achieved an induction time of 16.29 ± 0.14 h, representing the only sample to exceed the 13-h threshold mandated by ANP N°920/2023.^40^ These results suggest that combining the acidic extract with TBHQ achieves high oxidative stability while minimizing the required concentration of synthetic antioxidants

**Table 6:** Induction Times of Biodiesel Samples With TBHQ 100 ppm, Both Pure (control) and Antioxidant Extract Added

Sample	Induction time (h)
Biodiesel + TBHQ 100 ppm	5.00 ± 0.10
Biodiesel + alkaline extract (added by mechanical agitation) + TBHQ 100 ppm	3.11 ± 0.02
Biodiesel + alkaline extract (added by ultrasound agitation) + TBHQ 100 ppm	2.84 ± 0.04
Biodiesel + neutral extract, (added by mechanical agitation) + TBHQ 100 ppm	2.31 ± 0.06
Biodiesel + neutral extract (added by ultrasound agitation) + TBHQ 100 ppm	2.06 ± 0.01
Biodiesel + acidic extract (added by mechanical agitation) + TBHQ 100 ppm	16.29 ± 0.14
Biodiesel + acidic extract (added by ultrasound agitation) + TBHQ 100 ppm	9.60 ± 0.18

These results align with findings by De Souza et al.,^41^ who evaluated the oxidative stability of soybean biodiesel using bamboo leaf, potato peel, and lemon peel extracts, both individually and in combination with TBHQ. Individually, none of the natural extracts reached the 13-h induction time mandated by ANP N°920/2023,^40^ with the potato peel extract achieving the highest value (7.82 h). Furthermore, a TBHQ concentration of 1,000 ppm was required to reach an induction time of 12.32 h. Conversely, mixtures significantly enhanced stability at lower synthetic concentrations. For instance, combinations of 100 ppm TBHQ with 1,000 ppm bamboo leaf extract, 500 ppm potato peel extract, or 1,000 ppm lemon peel extract yielded induction times of 12.2, 13.44, and >13 h, respectively.^41^

Furthermore, a similar synergistic effect was reported by Boschen et al.,^28^ who found that a combination of 0.1% (w/v) citric acid and 11 g·L^–1^ of barley residue extract increased soybean biodiesel induction time to 14.67 ± 2.10 h. In contrast, the barley residue extract alone yielded 5.76 ± 0.11 h, and pure citric acid resulted in 5.46 ± 0.07 h. This significant enhancement in induction time indicates the presence of a synergistic mechanism.^28^

#### 2.3.1 Possible explanations for the antioxidant extracts’ performance

A possible explanation for the modest induction times of the extracts (Table 5) lies in the timing of their addition. While the acidic extract was introduced during the washing step after biodiesel production, the alkaline and neutral extracts were added directly during the transesterification reaction. Given the diverse acidity of potential antioxidant compounds, the KOH catalyst may have reacted with the acidic fraction of the extracts. This neutralization reaction would form insoluble organic acid salts, which are subsequently removed from the medium during the purification process.^29^

A further explanation for the modest performance of the alkaline and neutral extracts relates to the extraction conditions described in Section 4.2. Specifically, basic components such as KOH or methoxide, which are present in these solvents, likely induce the deprotonation of the extracted antioxidant compounds. By sequestering the protons, these basic species render the antioxidants unable to donate hydrogen to free radicals, thereby hindering their ability to inhibit biodiesel oxidation.^28^

Moreover, the superior performance of mechanical agitation, compared to ultrasonic processing, is likely attributable to the cavitation phenomenon inherent to the latter. In ultrasonic systems, localized pressure drops below vapor pressure, inducing the formation of cavitation bubbles. Upon collapse, these bubbles generate highly reactive hydroxyl radicals (·OH) through the dissociation of water and oxygen molecules. These radicals can react with, and subsequently degrade, the antioxidant compounds.^42,43^ Furthermore, the presence of dissolved oxygen during this cavitation process may accelerate the oxidative degradation of the biodiesel itself.

In addition, the combination of the acidic extract with 100 ppm of TBHQ yielded a higher induction time (16.29 ± 0.14 h) than the arithmetic sum of the induction times for both antioxidants individually (TBHQ 100 ppm = 5.00 ± 0.10 h; mechanically agitated acidic extract = 6.03 ± 0.04 h). Therefore, the most plausible explanation is a potentiation synergistic effect, which occurs when the combined activity of two or more substances exceeds the sum of their individual effects, in contrast to additive synergism.^44^ However, elucidating the exact synergistic mechanism remains notably challenging, primarily due to the complex, multicomponent nature of plant-based antioxidant extracts.^45–47^

### 2.4 Biodiesel physical chemical characterization

Table 7 presents the physicochemical properties for soybean biodiesel containing the acidic extract, added by mechanical agitation, which achieved an induction time of 16.29 ± 0.14 h. The sample exhibited a clear, light-yellow appearance, free of visible impurities. Its density (882 ± 0.2 kg·m^–3^) fell well within the range mandated by the ANP N°920/2023 established range (850 to 900 kg·m^–3^).^40^ This value is consistent with previous studies on soybean biodiesel, such as those by Özener et al.,^48^ (883.4 kg·m^–3^), Osarumwense et al.^49^ (877 kg·m^–3^), and Barbosa et al.^50^ (882.0 ± 0.6 kg·m^–3^).

**Table 7:** Biodiesel physical chemical characterization results

Analysis	Result	**Limit** ^a^,^b^	Unit	Methodology
Aspect	Translucent and free of impurities	Translucent and free of impurities^a^	-	Visual
Density at 20 °C	882 ± 0,2	850–900 ^[a]^	kg·m^–3^	ASTM D4052–18a
Flash point	123 ± 1,0	>100,0 ^[a]^	°C	ATSM D9318, proceeding C
Conductivity	341 ± 10,0	>25,0 ^[b]^	pS/m	ASTM D2624 and D4308

a Reference values specified in ANP Resolution n^0^ 798/2019 (biodiesel).^51^

b Reference values specified in ANP Resolution n^0^ 905/2022 (diesel).^52^

The biodiesel produced in this study presented a flash point value of 123 ± 1.0 °C, which is higher than the 100.0 °C minimum limit mandated by ANP N°920/2023.^40^ Interestingly, this result is identical to the flash point reported by Özener et al.^48^ (123 °C) for soybean biodiesel, and is consistent with the value reported by Osarumwense et al.^49^ (132 ± 4.1 °C).

Regarding conductivity, as ANP does not specify a limit for biodiesel, ANP Resolution N°905/2022 for petroleum diesel was adopted as a reference standard, which establishes a minimum conductivity of 25.0 pS/m.^52^ The biodiesel produced in this study exhibited a conductivity of 341 ± 10.0 pS/m, significantly exceeding this baseline and ensuring compliance with the cited safety and quality requirements.

### 2.5 Future Research Directions

The findings and limitations of this study suggest several avenues for future research. Among them, the addition of neutral and alkaline extracts during the post-transesterification washing phase should be evaluated to determine if this alternative application improves performance. Furthermore, high-performance liquid chromatography (HPLC) or gas chromatography–mass spectrometry (GC-MS) should be employed to quantify antioxidant recovery across different biodiesel production stages, specifically before and after both the transesterification reaction and washing steps. Such analyses would confirm the mechanisms of antioxidant loss, whether due to insolubility or proton sequestration, and provide a clearer picture of radical distribution in the final product.

Additionally, Fourier-transform infrared (FTIR) spectroscopy should be employed to detect potential salt formation in neutral and alkaline extracts post-transesterification. This would involve identifying characteristic shifts in carbonyl or hydroxyl stretching frequencies, which serve as indicators of reaction products with the KOH catalyst. Furthermore, quantifying the peroxide value of the biodiesel treated with these extracts would provide a more comprehensive assessment of their susceptibility to primary oxidation, offering deeper insights into the induction time results.

Furthermore, techniques such as electron paramagnetic resonance (EPR) spectroscopy and HPLC could be employed in subsequent studies to monitor cavitation-induced hydroxyl radicals (·OH), their subsequent reactions with antioxidants, and the specific role of dissolved oxygen in accelerating biodiesel oxidation. Additionally, UHPLC analysis utilizing a broader range of analytical standards would facilitate a more comprehensive characterization of the extract compositions. Finally, further research is highly recommended to elucidate the precise molecular mechanisms driving the synergistic effect observed between the acidic extract and TBHQ, potentially revealing new pathways for oxidative stabilization.

Moreover, a dedicated stability study is recommended to evaluate the behavior of the antioxidant extracts during prolonged storage. Should degradation be observed over time, nanoencapsulation could be employed to enhance the extracts’ stability and provide a controlled-release delivery system for the biodiesel. This approach would potentially mitigate rapid oxidation and extend the shelf life of the final fuel product

Furthermore, future research should investigate the behavior of these antioxidant extracts at larger scales. This study was conducted on a laboratory scale, and unforeseen challenges may arise during process scale-up. Notably, 300 mL of acidic extract and 0.1 g of TBHQ were required to increase the soybean-based biodiesel induction time to 16.29 ± 0.14 h. Specifically, 31.8 g of RTSS and 300 mL of 0.5% (v/v) HCl were utilized to produce this volume of antioxidant extract. Additionally, a detailed characterization of the RTSS composition is recommended as the presence of trace metallic impurities, such as copper and iron ions, may potentially catalyze biodiesel oxidation and decrease the overall induction time, thereby necessitating further purification strategies.

Finally, while this study focused on soybean-based biodiesel, future research should investigate the performance of these antioxidant extracts across a broader range of feedstocks. Comparative analyses using various synthetic antioxidants, varying concentrations, and diverse storage temperatures are essential to validate these findings and assess the robustness of the extraction method across different biodiesel systems.

## 3 Conclusions

The best antioxidant activity results were obtained with the optimized acidic medium extract of RTSS, extracted for 60 min and with a seed shell concentration of 106 g·L^–1^. This extract presented an antioxidant activity of 79.664% and a total phenolic compound concentration of 761.15 ± 116.23 mg GAE·100 g^–1^.

Furthermore, evaluating the extracts’ antioxidant effect on soybean biodiesel via induction time showed that the optimized acidic extract, when added during the washing stage by mechanical agitation and combined with 100 ppm of the synthetic antioxidant TBHQ, yielded an induction time of 16.29 ± 0.14 h. This value is greater than the 13-h minimum stipulated by ANP N°920/2023.^40^ These results indicate a significant synergistic effect between the natural acidic extract and the synthetic antioxidant in inhibiting the oxidative degradation of soybean-based biodiesel.

Finally, although TBHQ is a synthetic antioxidant, its application is minimized to a concentration of 100 ppm. Conversely, the acidic medium extract of the RTSS is a natural antioxidant derived from a renewable agroindustrial byproduct. While the acidic extract did not meet regulatory requirements independently, it acts as a potent coantioxidant, and its synergistic behavior with 100 ppm TBHQ provides a possible strategy to stabilize soybean biodiesel. This approach aligns with the biorefinery concept, which leverages the rubber tree seed’s potential for high-value output, including biodiesel, ethanol, and biobased additives.

## 4 Materials and Methods

### 4.1 Feedstock Acquisition

Samples of *Hevea brasiliensis* seeds were collected in Paranaíba, Mato Grosso do Sul, Brazil, by the company Kaiser Agro Florest, and subsequently transported to UNICENTRO, in Guarapuava, Paraná, Brazil. The seeds were dehulled, and the kernels were discarded. The remaining shells were then processed in a high-speed blender to achieve a uniform particle size, thereby facilitating the subsequent extraction process.

It is worth noting that this research was registered in SisGen (National System for the Management of Genetic Heritage and Associated Traditional Knowledge) under registration number A4C9E3E.

### 4.2 Extract Preparation

Extraction was performed in three distinct media: alkaline, neutral, and acidic. For the alkaline medium assays (pH 14), a solvent mixture was prepared consisting of 1.5% potassium hydroxide (KOH) and 30% methanol (v/v), relative to the total volume of soybean oil intended for transesterification. RTSS was then added to this solution at concentrations of 80, 100, and 120 g·L^–1^, with extraction times of 30, 60, and 90 min followed by filtration. A similar procedure was adopted for the neutral medium (pH 7), utilizing 30% methanol (v/v), relative to the total volume of soybean oil intended for transesterification, but without the addition of KOH.^53^ However, based on preliminary tests which indicated lower antioxidant yields at shorter times, the neutral assays were conducted using higher RTSS concentrations (140, 160, and 180 g·L^–1^) and extended extraction times (120, 150, and 180 min).

Meanwhile, for the acidic medium (pH = 1), the RTSS were added, in concentrations of 90, 100, and 110 g·L^–1^, to a 0.5% (v/v) hydrochloric acid (HCl) solution for 60, 90, and 120 min. As before, lower values were insufficient to reach the maximum antioxidant activity. Finally, the solution containing the extracted antioxidants was filtered.^53^

The optimization of the extraction processes was conducted using a 3^2^ full factorial experimental design. The experimental factors, including both actual and coded levels, are summarized in Table 8.

**Table 8:** 3^2^ Full Factorial Design for the Optimization of the Extraction Processes, with Codified and Respective Actual Values

codified values		atual values for alkaline medium		actual values for neutral medium		actual values for acidic medium	
time	concentration	time (min)	concentration (g·L^–1^)	time (min)	concentration (g·L^–1^)	time (min)	concentration (g·L^–1^)
–1	–1	30	80	120	140	60	90
–1	0	30	100	120	160	60	100
–1	1	30	120	120	180	60	110
0	–1	60	80	150	140	90	90
0	0	60	100	150	160	90	100
0	1	60	120	150	180	90	110
1	–1	90	80	180	140	120	90
1	0	90	100	180	160	120	100
1	1	90	120	180	180	120	110

### 4.3 Extract Antioxidant Activity Quantification

To determine the total antioxidant activity, the DPPH (2,2-diphenyl-1-picrylhydrazyl) free radical capture method was used. In this method, DPPH is reduced to DPPH-H (2,2-diphenyl-1-picrylhydrazine) in the presence of an antioxidant compound. As a result, the solution color changes from violet to yellow and the absorbance at 515 nm decreases.^54^

More specifically, a 0.1 mmol L^–1^ solution of DPPH in ethanol, referred to as the stock solution, was prepared and further diluted in ethanol by a factor of 8. Next, a mixture containing 2.7 mL of the diluted DPPH solution and 0.3 mL of the solvent used to prepare the extract (A_control_) was prepared, and its absorbance at 515 nm was measured in a Shimadzu UV 1800 UV/vis spectrophotometer. This procedure was repeated for the samples, but, instead of the solvent, 0.3 mL of the extract in question (A_sample_) was added and was allowed to react for 30 min before the absorbance measurement. For the blank, pure extract and ethanol were used, the latter being used to obtain the final absorbance of the DPPH reduction alone. All tests were performed in triplicate, and the results were expressed as a percentage (antioxidant activity, or %AA) of DPPH free radical capture, calculated based on eq 4.

$$\%\mathrm{AA}=[(A_{\mathrm{control}}-A_{\mathrm{sample}}-A_{\mathrm{blank}})/A_{\mathrm{control}}]\times 100\%$$ 4

where A_control_ represents the DPPH control solution absorbance and A_sample_ represents the DPPH’s absorbance with the respective sample.

### 4.4 Total Phenolic Compound Quantification

The quantification of total phenolic compounds was performed according to the adapted methodology described by Oleinik et al.,^18^ in which 2500 μL of 0.1 mol·L^–1^ Folin Ciocalteau solution and 2000 μL of saturated sodium carbonate (Na_2_CO_3_) solution were added to 100 μL of each extract, and the assays were then left for 1 h. Then, the assay’s absorbance was measured in triplicate at 720 nm, against a water blank.

To obtain an analytical curve, gallic acid was used in concentrations ranging from 0.1 to 4.0 mg·L^–1^, and the total phenolic content was expressed in mg of gallic acid equivalents per 100 g^–1^ of dry sample (mg GAE·100 g^–1^).^18^

### 4.5 Phenolic Compound Profiles via UHPLC

The phenolic compound profiles were determined with a Dionex UltiMate 3000 (Thermo Fisher Scientific) UHPLC chromatograph equipped with a DAD-3000RS UV–visible detector, a LPG-3400SD quaternary pump, a TCC-3400RS oven and a 20 μL manual injection system. This instrument was installed in the Federal University of Technology of Paraná (UTFPR) Analytical Center, in Toledo, Paraná, Brazil. Furthermore, a ZORBAX Eclipse XDB C18 column (250 mm × 4.6 mm, 5 μm) was used as the stationary phase, at a fixed temperature of 40 °C, and a mixture of 0.5% acetic acid acidified ultrapure water (A) with 0.5% acetic acid acidified methanol (B) was used as the mobile phase, with a 1 mL/min flow rate and in a gradient elution, as specified in Table 9. Moreover, the detection was carried out at 280 nm, also with monitoring at 295, 265, and 360 nm, and the following phenolic compound standards were used: chlorogenic acid, p-coumaric acid, caffeic acid, ascorbic acid, gallic acid, quercetin, ferulic acid, curcumin, rutin, kaempferol, cinnamic acid, ellagic acid, bisdemethoxycurcumin, demethoxycurcumin and catechin.

**Table 9:** Mobile Phase Gradient for the UHPLC Determination of Phenolic Compounds

time (min)	% A	% B
0	85	15
40	35	65
45	5	95
50	5	95
55	85	15
60	85	15

### 4.6 Statistical Analysis

In order to optimize the extraction process, experimental designs were generated, considering the concentrations and extraction times of the RTSS as independent variables. Meanwhile, the percentage of DPPH capture (%AA) was considered as the response variable. The validation and analysis of the statistical results were carried out using response surface methodology using the Statistica software.^55^ The assays corresponding to the best extraction conditions for each solvent were added to biodiesel samples.

The validation of the resulting mathematical models consisted of analyses of residual dispersion graphs, Pareto graphs, and analysis of variance (ANOVA) tables. Considering the ANOVA data, the percentages of variation (R) and maximum explained variation (R^2^) were calculated, as well as the F values for regression (F-ratio) and lack of fit (F_LoF_). Furthermore, experimental validation was conducted at specific points within the design space. The consistency between predicted and experimental results was confirmed using a single-sample *t* test.^20^

### 4.7 Biodiesel Production

In order to produce biodiesel, soybean oil was transesterified with methanol and KOH as a catalyst in a ratio of 1:0.3:1% v/v/m, respectively. First, the soybean oil was heated to 80 °C and the mixture of methanol and KOH was heated to 40 °C to solubilize the catalyst. Then, the methanol and KOH solution was added to the oil and the temperature was maintained at 60 °C under constant stirring for 1 h. Later, this mixture was transferred to a decantation funnel for 24 h to separate the biodiesel and glycerin.^56^

Subsequently, biodiesel was washed in three stages: first with 0.1% HCl, then with sodium chloride solution (NaCl) and, finally, with water. The washing processes were carried out in a decantation funnel for 24 h and the washing solution accounted for 30% (v/v) of the total volume of biodiesel. Finally, the biofuel was dried in an oven at 110 °C for 3 h.^57^

### 4.8 Addition of the Antioxidant Extracts to Biodiesel

The acidic antioxidant extracts were added to the biodiesel during the initial washing stage, and the resulting mixture was homogenized via ultrasonication or mechanical agitation. Specifically, the acidic extract replaced the 0.1 M HCl solution, added at a concentration of 30% (v/v) relative to the total biodiesel volume. Subsequent washing steps were performed as standard, using NaCl solution and water.

For the alkaline and neutral variants, the antioxidant solutions were added during the transesterification reaction.^28^ Specifically, the neutral extract replaced methanol at 30% (v/v) of the total biodiesel volume, with the KOH catalyst added at a 1.0% (m/m) biodiesel-to-KOH ratio. Similarly, the alkaline extract served as a substitute for both methanol and KOH, again accounting for 30% (v/v) of the biodiesel volume.

### 4.9 Evaluation of the Antioxidant Effect over Biodiesel

In order to evaluate the antioxidant extracts’ effectiveness when added to biodiesel, the induction time (IT), which must be at least 13 h, according to ANP N°920/2023 standard,^40^ was used as a parameter. Moreover, the IT readings were obtained using a Metrohm Rancimat 873 device at a temperature of 110 °C, with an air insufflation rate of 10 L/h, following Standard EN 14112:2016.^58,59^

Finally, the optimal extraction method was selected based on a multicriteria approach, prioritizing both the highest observed antioxidant activity and the maximum enhancement of the biodiesel induction time.

### 4.10 Biodiesel Physical Chemical Characterization

The analyses listed below were performed according to the following references or standards: oxidation stability at 110 °C (EN 14112:2016),^59^ acidity index (ASTM D664–18e2 – Method B),^60^ specific gravity at 20 °C (ASTM D4052–18a),^61^ flash point (ASTM D93–18 Procedure C),^62^ conductivity (ASTM D1125),^63^ and sulfur content (ASTM D5504).^64^

## Supplementary Material



## Notes

Pareto charts for the alkaline, neutral, and acidic medium extraction mathematical models (Figures S1, S3, and S5); graphics of raw residuals vs Observed values for the mathematical models (Figures S2, S4, and S6); UHPLC Chromatograms for the alkaline, neutral, and acidic medium extracts (Figures S7–S9); tables detailing validation for the mathematical models (Tables S1–S3) (PDF)

## Abbreviations

DPPH: 2,2-diphenyl-1-picrylhydrazyl
TBHQ: tertiary butylhydroquinone
ANP: Brazilian National Petroleum Natural Gas and Biofuels Agency
BHT: butylated hydroxytoluene
TBHQ: *tert*-butylhydroquinone
BHA: butylated hydroxyanisole
PY: pyrogallol
PG: propyl gallate
RTSS: rubber tree seed shells
GAE: gallic acid equivalents
DPPH-H: 2,2-diphenyl-1-picrylhydrazine
%AA: antioxidant activity
IT: induction time
