# Supplementary material for: Alkaline, Neutral and Acidic Extracts from Rubber Tree (Hevea brasiliensis) Seed Shells and Their Application as Biodiesel Antioxidants
Source: ACS Omega. 2026 May 11;11(20):30025–35. doi: 10.1021/acsomega.6c01549 (PMC13216911; doi:10.1021/acsomega.6c01549)
Supplement: Supplementary file 1 [file ao6c01549_si_001.pdf]

Alkaline, neutral and acidic extracts from rubber tree (*Hevea brasiliensis*) seed shells and their application as biodiesel antioxidants

Supporting Information

Giovanna Oleinik,[1] Giovano Tochetto,[1] Letiére C. Soares,[2] Fernanda O. Lima,[2] Dalila M. Benvegnú,[2] Gabrielle C. Peiter,[3] André L. Gallina*,[1]

affiliations:

[1] Department of Chemistry, Midwestern Paraná State University (UNICENTRO), Alameda Élio Antonio Dalla Vecchia, 838, Center for Technological Development of Guarapuava, Paraná (PR) 85040-167, Brazil

[2] Federal University of Fronteira Sul (UFFS), *Campus* Realeza, Postal Office Box 253, Avenida Edmundo Gaievski, 1000, Rodovia BR 182 – Km 466, Realeza, Paraná (PR) 85770-000, Brazil.

[3] Graduate Program in Chemical and Biotechnological Processes, Federal University of Technology - Paraná (UTFPR), Rua Cristo Rei, 19, Toledo, Paraná (PR) 85902-490, Brazil.

* Corresponding author. E-mail: andregallina@unicentro.br

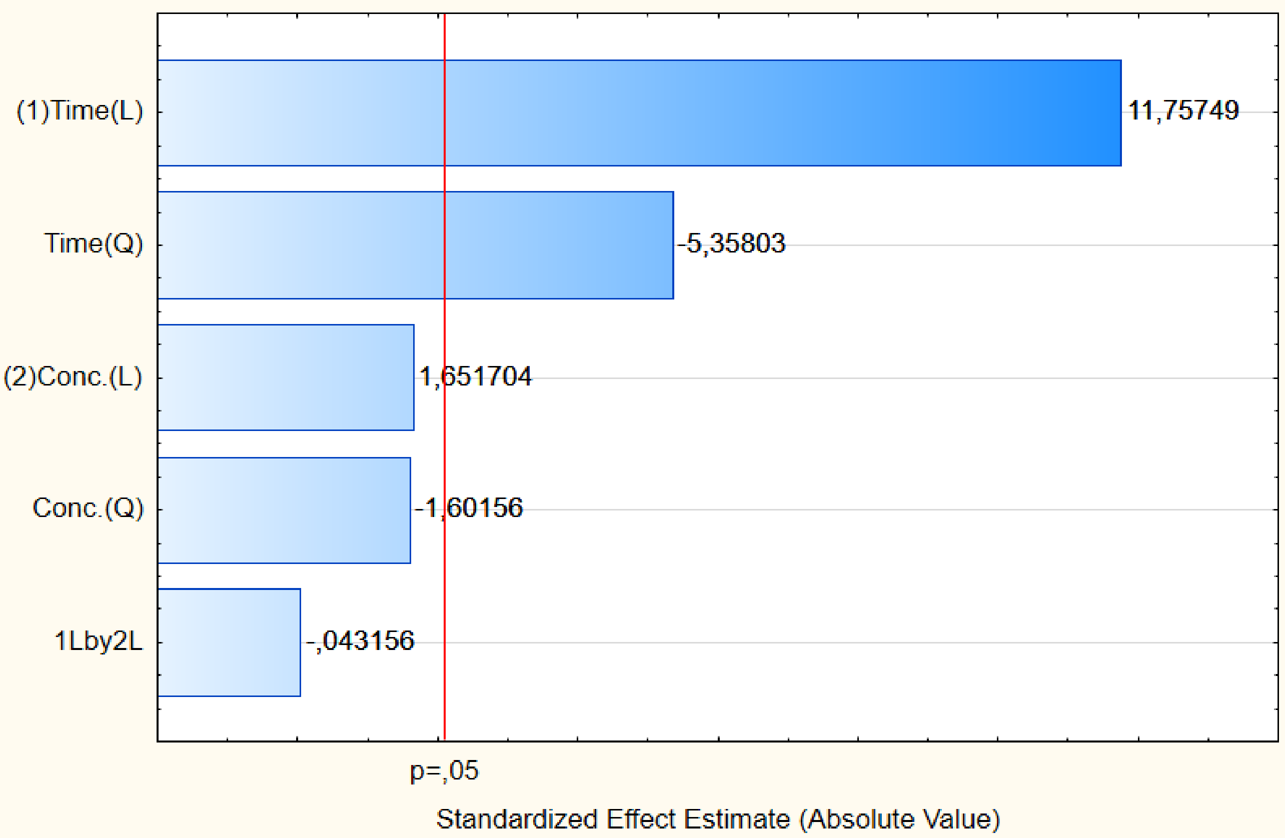

Figure S1: Pareto chart of standardized effects for the alkaline medium extraction quadratic model

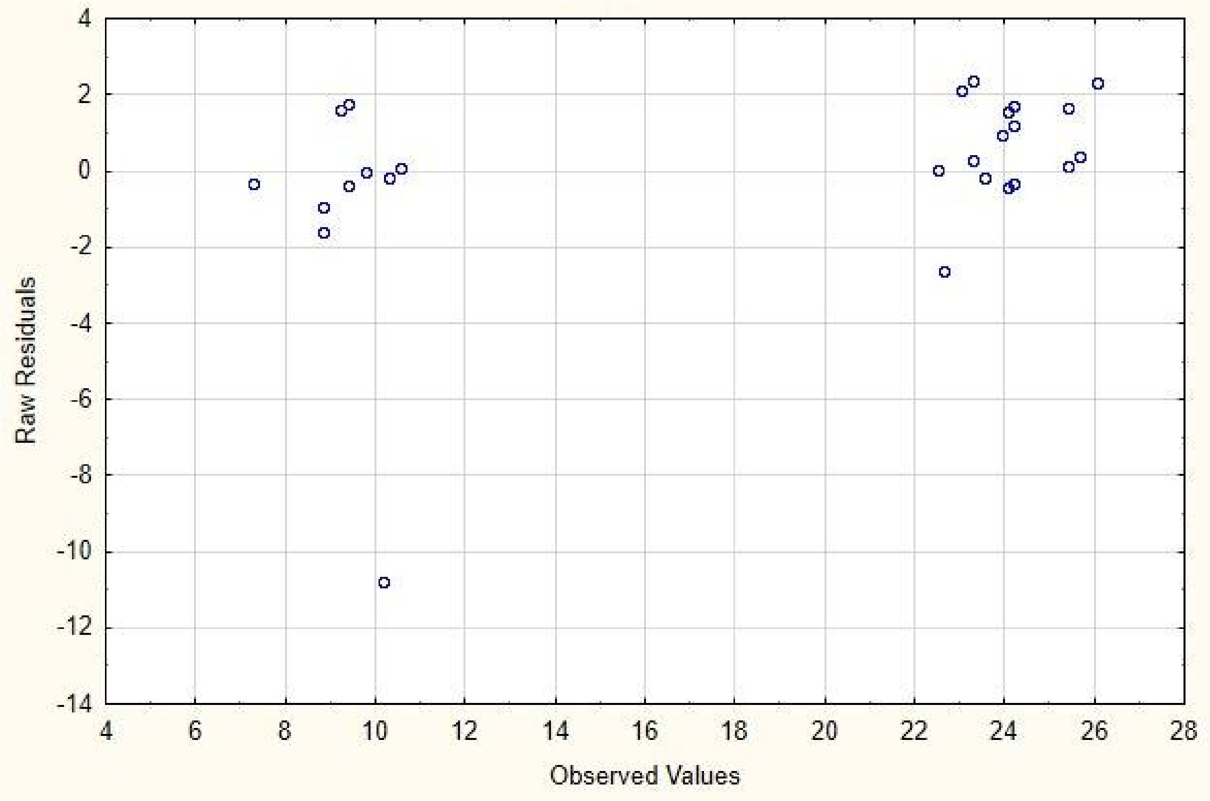

Figure S2. Raw residuals vs. Observed values for the alkaline medium extraction mathematical model

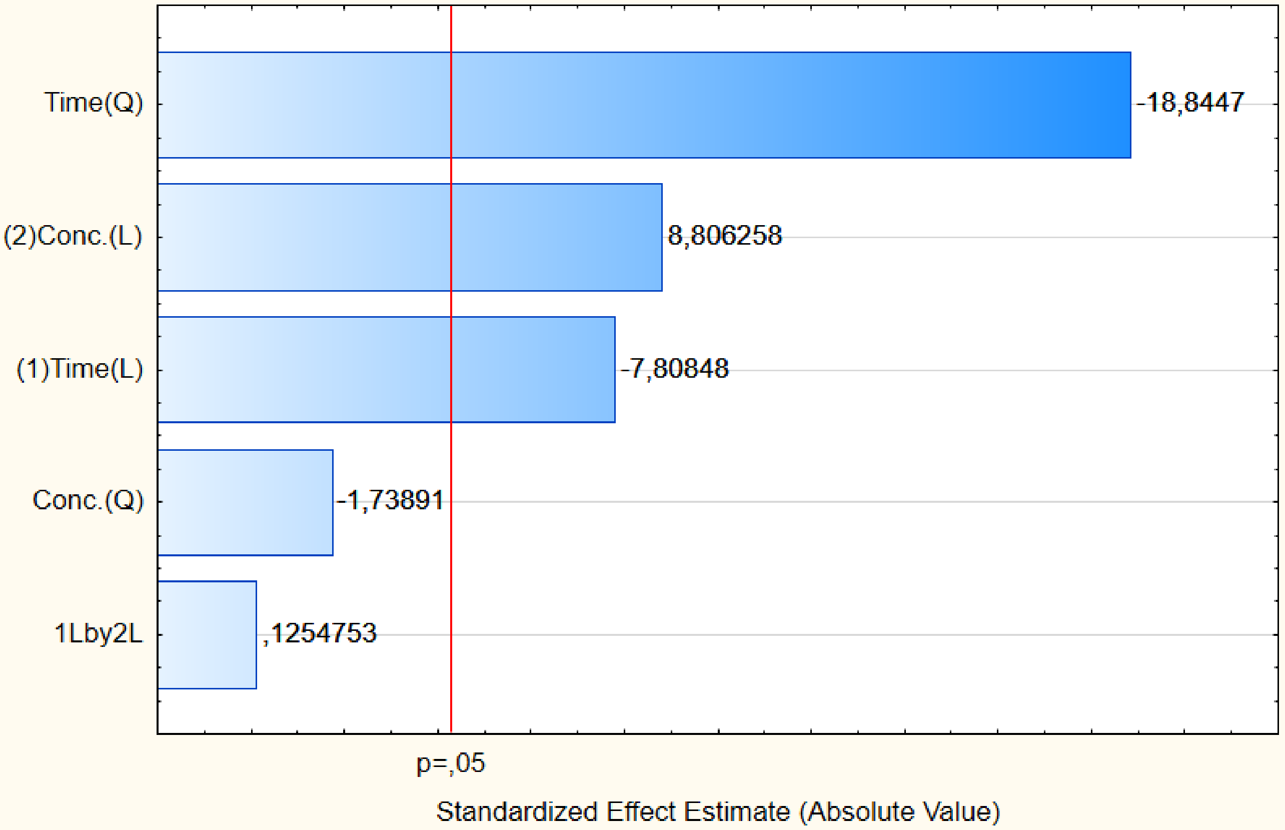

Figure S3: Pareto chart of standardized effects for the neutral medium extraction quadratic model

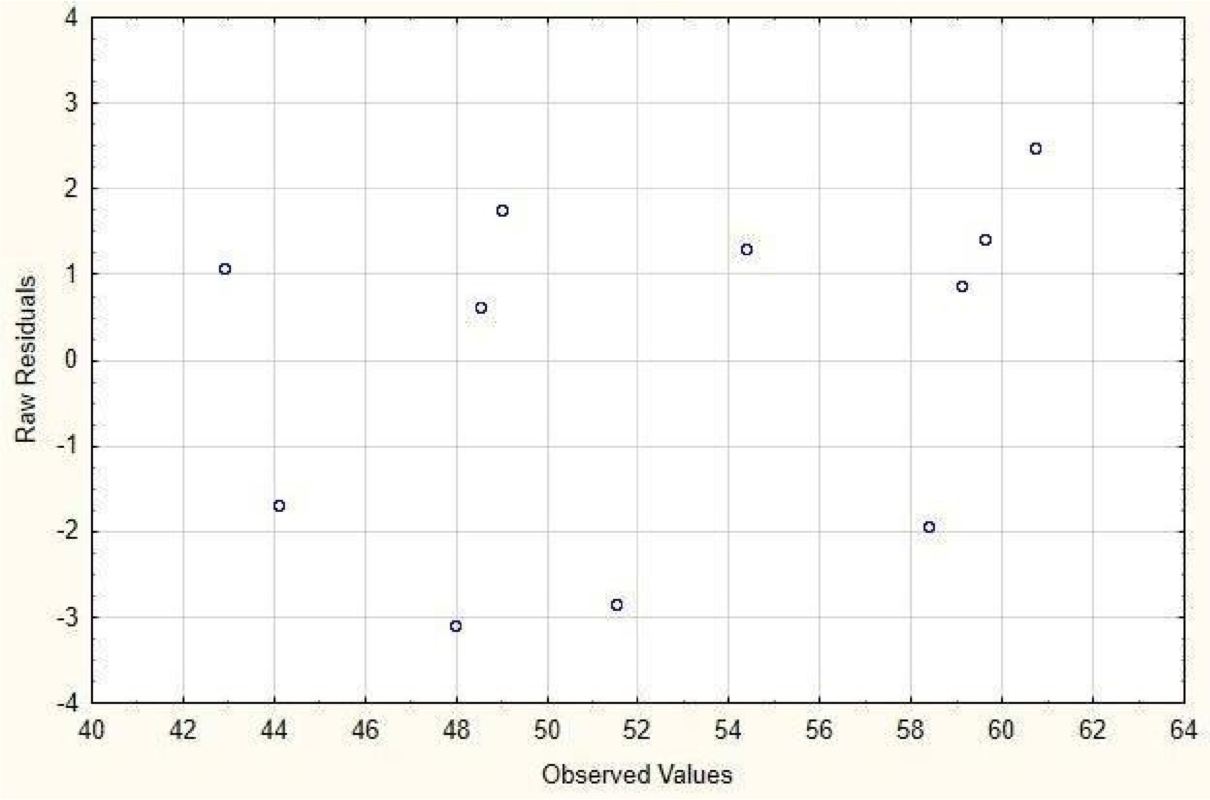

Figure S4. Raw residuals vs. Observed values for the neutral medium extraction mathematical model

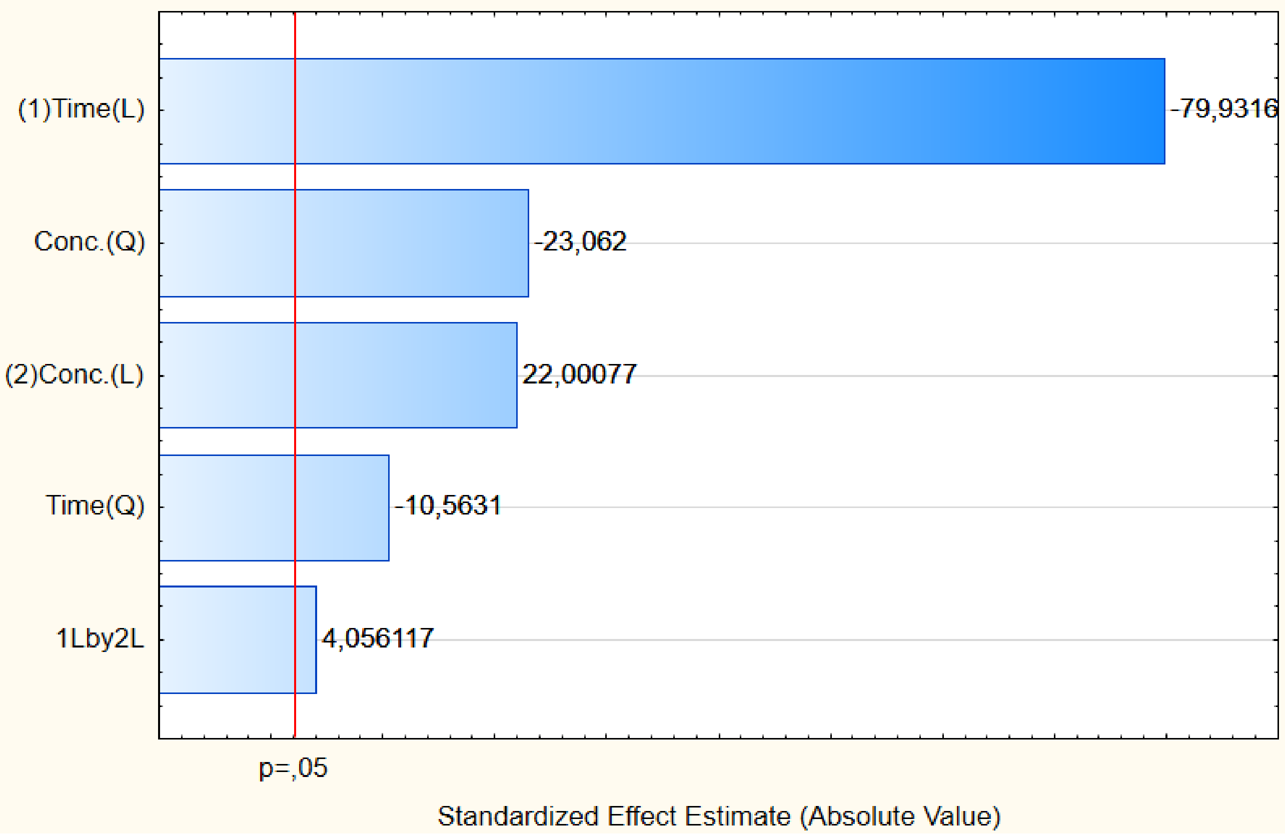

Figure S5: Pareto chart of standardized effects for the acidic medium extraction quadratic model

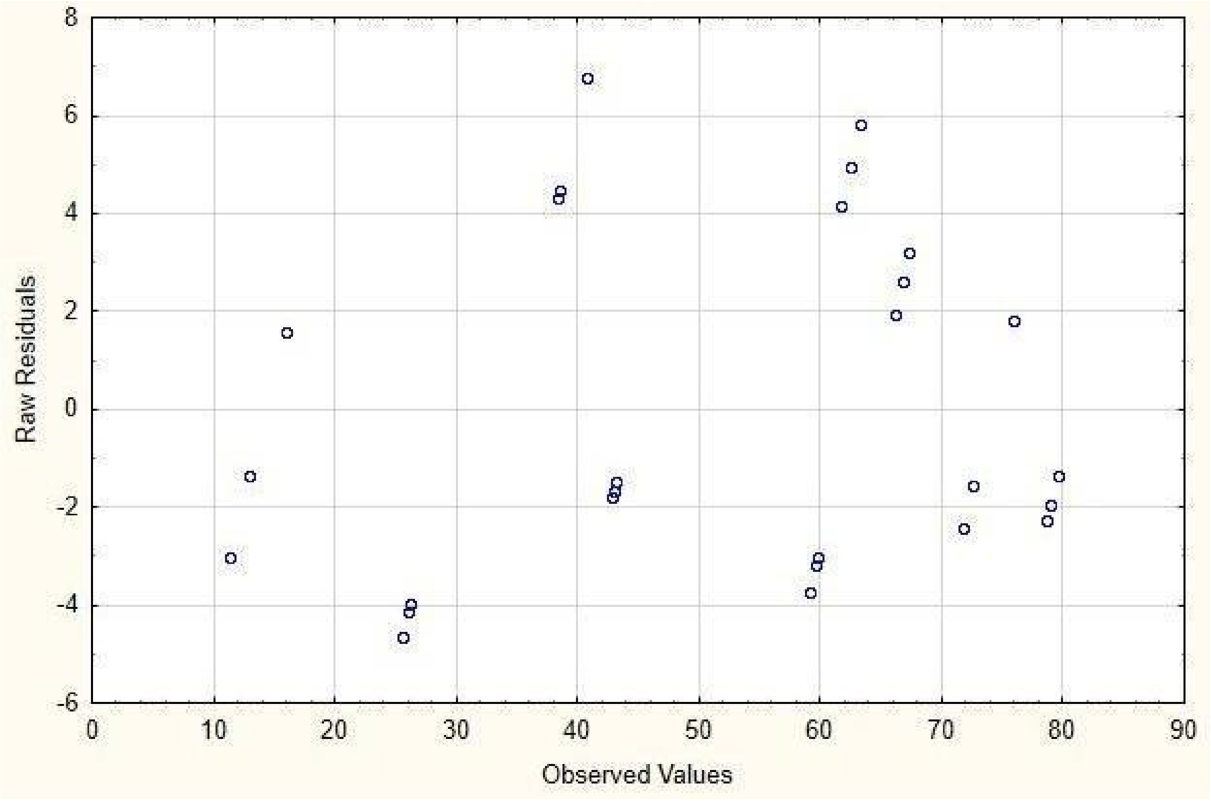

Figure S6. Raw residuals vs. Observed values for the acidic medium extraction mathematical model

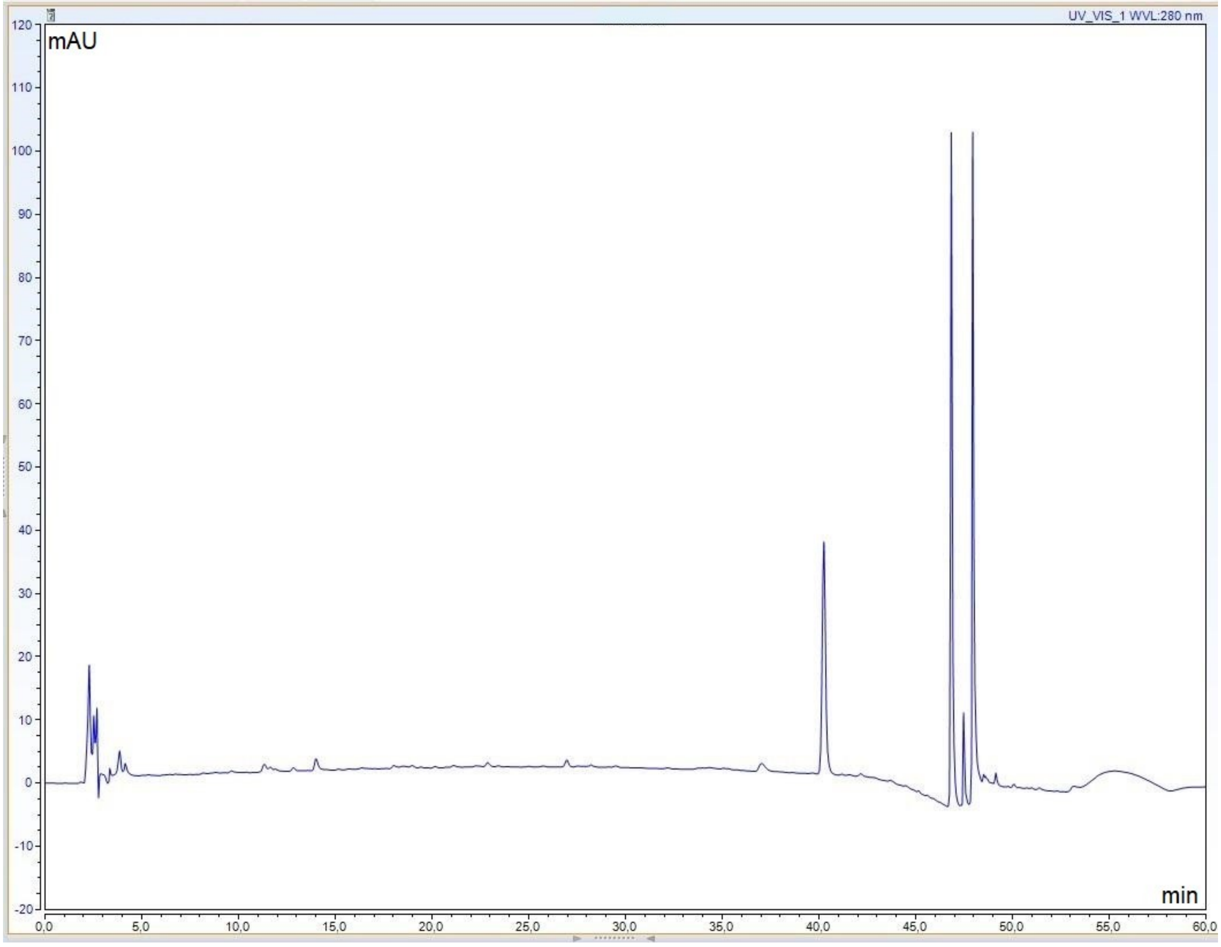

Figure S7: UHPLC chromatogram of phenolic compounds of the alkaline extract. Wavelength: 280 nm.

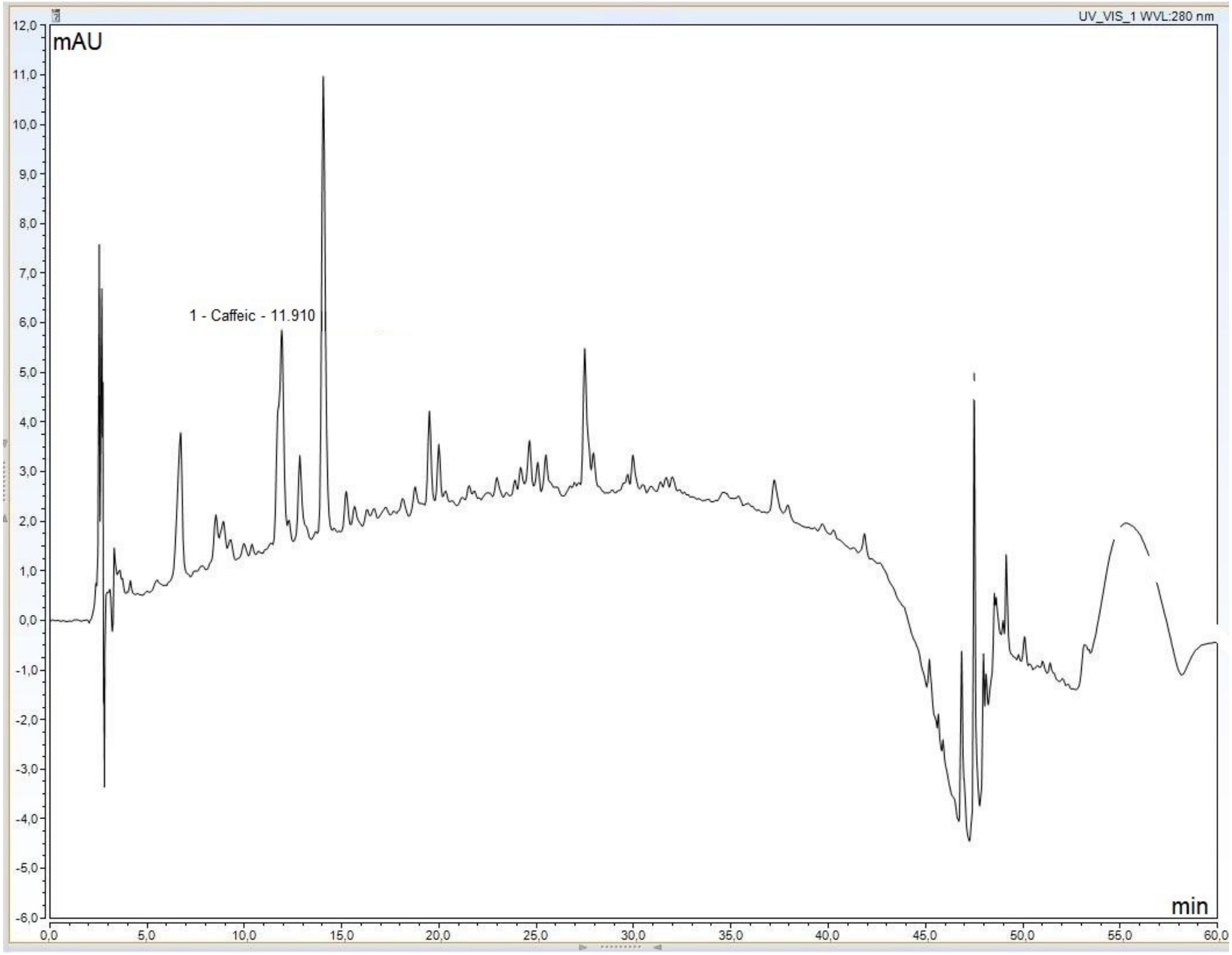

Figure S8: UHPLC chromatogram of phenolic compounds of the neutral extract. Wavelength: 280 nm.

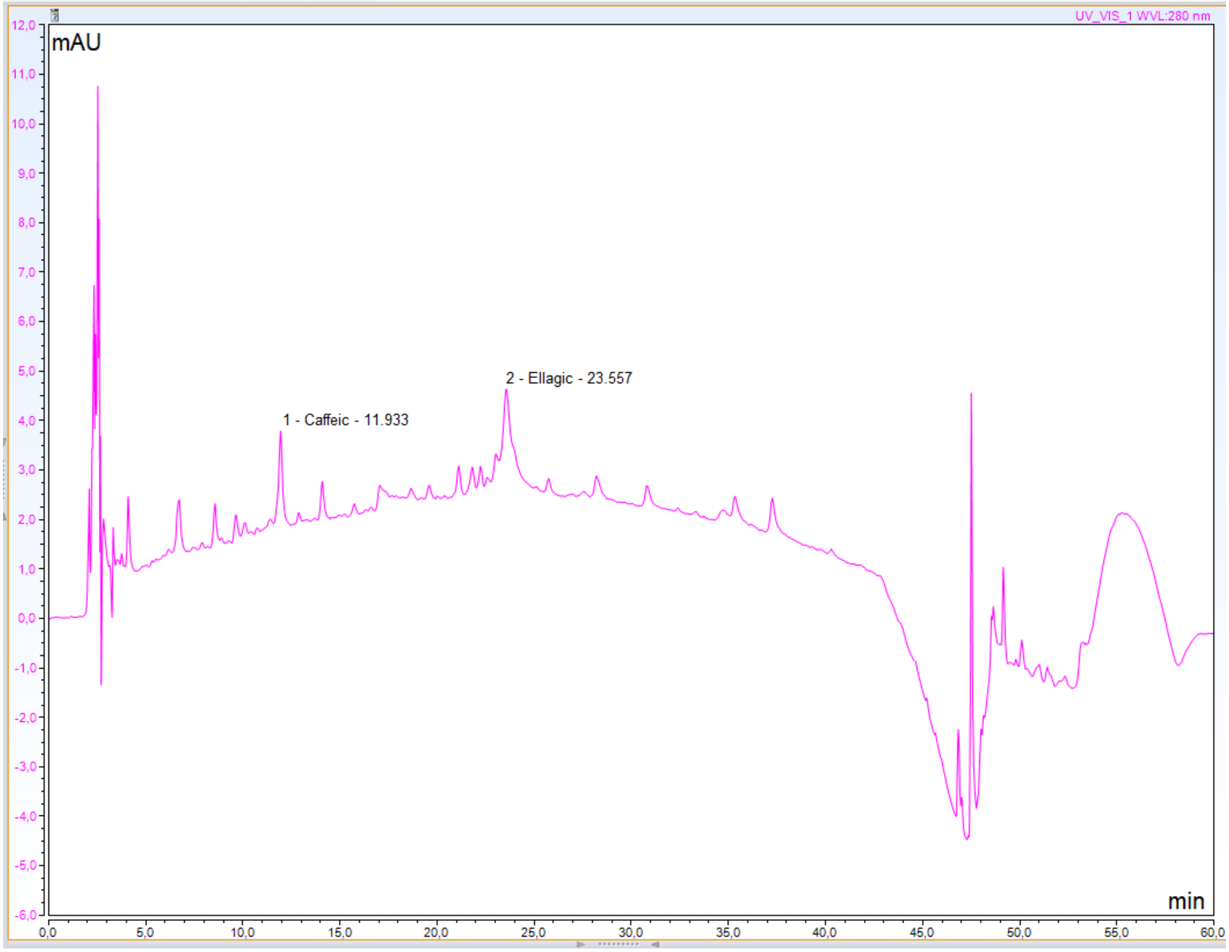

Figure S9: UHPLC chromatogram of phenolic compounds of the acidic extract. Wavelength: 280 nm.

Table S1: Comparison of predicted and experimental values for the validation of the alkaline medium quadratic model

Codified Time	Concentration	Actual Time (min)	Actual Concentration ($g.L^{-1}$)	Predicted result (%)	Experimental result (%)	Experimental Mean (%)	t-value	p-value
0.6	0.6	78	112	26.048	27.77			
0.6	0.6	78	112	26.048	27.45	27.453 ± 0.313	7.771	0.016
0.6	0.6	78	112	26.048	27.14			

Table S2: Comparison of predicted and experimental values for the validation of the neutral medium quadratic model

Codified Time	Concentration	Actual Time (min)	Actual Concentration ($g.L^{-1}$)	Predicted result (%)	Experimental result (%)	Experimental Mean (%)	t-value	p-value
-1	1	120	180	53.07	55.01	54.38 ± 0.56	-4.026	0.056
-1	1	120	180	53.07	53.93			
-1	1	120	180	53.07	54.20			

Table S3: Comparison of predicted and experimental values for the validation of the acidic medium quadratic model

Codified Time	Concentration	Actual Time (min)	Actual Concentration ($g.L^{-1}$)	Predicted result (%)	Experimental result (%)	Experimental Mean (%)	t-value	p-value
-1	1	60	110	74.16	71.72			
-1	1	60	110	74.16	72.59	73.44 ± 2.26	-0.554	0.635
-1	1	60	110	74.16	76.00			
